# Aflibercept Suppression of Angiopoietin-2 in a Rabbit Retinal Vascular Hyperpermeability Model

**DOI:** 10.1167/tvst.12.10.5

**Published:** 2023-10-06

**Authors:** Claudia Lange, Reimo Tetzner, Tobias Strunz, Kay D. Rittenhouse

**Affiliations:** 1 Research & Development, Precision Medicine Markers, Bayer AG, Berlin, Germany; 2 Research & Development, Biosample Operation Management and Assay Technologies, Bayer AG, Berlin, Germany; 3 Research & Development Biomedical Data Science II, Bayer AG, Wuppertal, Germany; 4 Formerly at Medical Affairs, Bayer Consumer Care AG, Basel, Switzerland

##  

In our recent publication, we demonstrate an effect of vascular endothelial growth factor (VEGF) impacting angiopoietin-2 (ANG2) levels using a rabbit retinal vascular hyperpermeability model. After intra-vitreal challenge with human VEGF, ANG2 levels significantly increased in two ocular tissues, the vitreous humor and retina. Additionally, elevated ANG2 levels were significantly reduced by anti-VEGF agents to normal protein levels in the vitreous and messenger RNA (mRNA) levels in the retina.^1^ Comparable findings are reported using an oxygen-induced retinopathy model in mice.^2^ Rojo Arias et al.^2^ reported an increase of ANG2 expression in response to hypoxia, as well as a consistent down-regulation of ANG2 expression after treatment with aflibercept.

The VEGFR signaling pathway is the master regulator of pathologic angiogenesis.^3^ Inhibition of VEGF is a well-established mechanism of action to treat retinal diseases such as neovascular age-related macular degeneration (nAMD), diabetic macular edema (DME), and retinal vein occlusion. It is also well established that the use of anti-VEGF agents as treatment can provide favorable, clinically significant outcomes for patients. In contrast to VEGF inhibition, the clinical relevance of ANG2 inhibition in patients with retinal disease, such that levels are suppressed to below physiologically normal levels, has yet to be demonstrated. Clinical trials for nesvacumab, a fully human anti-ANG2 antibody^4^ coadministered with aflibercept, failed to demonstrate superiority in gains in visual acuity in both nAMD (ONYX)^5^ and DME (RUBY),^6^ and anatomical outcomes were not considered clinically meaningful. The results of our study, which support the hypothesis that ANG2 suppression may occur secondarily following treatment with anti-VEGF agents, is in line with the US Food and Drug Administration's (FDA's) viewpoints on the clinical relevance of the ANG2 pathway in nAMD and DME. The FDA reviewers stated in the faricimab drug approval package, “[Nesvacumab] trial results available publicly raise questions about the contribution of Ang-2 inhibition when in combination with anti-VEGF inhibition” and that “the contribution of Ang-2 inhibition to the treatment effect and clinical response for nAMD and DME has yet to be established.”^7^^,^^8^

In addition to the observations regarding ANG2 in our study, comparable results were observed for mRNA levels of platelet-derived growth factor subunit B (PDGFB), as they were supressed after treatment with anti-VEGF agents in the retina.^1^ Similarly, recent clinical trials examining dual VEGF and either PDGFB receptor (CAPELLA) or PDGFB (FOVISTA) inhibition failed to meet their primary endpoints of superior improvements in visual acuity in patients with nAMD compared to patients receiving anti-VEGF agents alone.^9^^,^^10^ These observations support the hypothesis that VEGF is the main pathologic driver in retinal diseases and that selective inhibition of mediators downstream of VEGF activation does not provide additional clinical benefit compared to the use of anti-VEGF agents alone.

In their Letter to the Editor, Avery et al. describe ANG2 levels in aqueous humor (AH) of patients with age-related macular degeneration (AMD) and DME prior to treatment as well as 1, 4, and 8 weeks after treatment with faricimab or aflibercept. The lower limit of quantification (LoQ) is reported as 4.04 pg/mL, and median levels prior to treatment for AMD and DME are reported as 5 to 6 pg/mL and 8 to 9 pg/mL, respectively. Bogman et al.,^11^ referring to the same clinical studies and using the same assay, reported no detectable ANG2 levels prior to treatment in 40% of patients with AMD and 26% of patients with DME. In addition, the detected ANG2 levels reported are considerably lower compared to previously published levels.^12^^,^^13^ For example, Ng et al.^12^ reported ANG2 levels of 9.44 pg/mL in healthy control AH samples and up to 7-fold higher ANG2 levels in AH of untreated AMD patients (42.88 pg/mL). Nevertheless, in the course of treatment with aflibercept, ANG2 AH median levels appear to decrease over time, and the number of individuals showing ANG2 levels below the LoQ increased (Letter to the Editor, Avery et al.), despite the fact that aflibercept does not directly bind to ANG2. These observations are consistent with results reported by Lange et al.,^1^ where it was shown that treatment with anti-VEGF agents leads to the reduction of elevated ANG2 levels to normal levels.

Furthermore, the claim of durability of ANG2 suppression by faricimab through week 16 is not well supported, as only limited data up to week 8 are included in Figure 1. The claim of durability is also in conflict with data reported by Bogman et al.,^11^ who reported increasing median levels of ANG2 in the AH at 12 and 16 weeks after treatment with faricimab. This effect is even more pronounced considering the high amount of outliers throughout all time points. Notably, the presented time points do not reflect a real time course of one cohort of patients but were generated using a merged data set from different clinical studies. This results in heterogeneous patient sampling, and the shown time points may not necessarily be related to the first dose. Overall, it is challenging to evaluate the data and interpretation from Avery et al. because no statistical analysis was reported.

Although our study was conducted using a nonclinical approach, we deemed it necessary to discuss and to contextualize the findings and their potential clinical relevance, since active agents of marketed products, Eylea (aflibercept) (Bayer AG, Leverkusen, Germany/Regeneron Pharmaceuticals, Inc., Tarrytown, NY, US), Lucentis (ranibizumab) (Novartis Pharma GmbH, Nuremberg, Germany/Genentech, Inc., South San Francisco, CA, US), and Beovu (brolucizumab) (Novartis Pharma GmbH, Nuremberg, Germany/Novartis Pharmaceuticals Corporation, East Hanover, NJ, US), have been investigated. Additionally, the anti-ANG2 effects of aflibercept, ranibizumab, and brolucizumab must be carefully interpreted, given that the additional clinical relevance and benefit of suppressing ANG2 over the confirmed anti-VEGF activity has yet to be demonstrated.^8^

In summary, we see no conflict in the data of Avery et al. with our results obtained in the rabbit model. The secondary effect of VEGF inhibition on ANG2 seems to be similarly indicated in their clinical data, as ANG2 levels are reduced and some appear to fall even below detectable levels in a large proportion of patients after treatment with aflibercept.
